# Stem cells, quiescence and rectal carcinoma: an unexplored relationship and potential therapeutic target

**DOI:** 10.1038/bjc.2011.362

**Published:** 2011-09-20

**Authors:** S Buczacki, R J Davies, D J Winton

**Affiliations:** 1 Cancer Research UK, Cambridge Research Institute, Li Ka Shing Centre, Robinson Way, Cambridge CB20RE, UK; 2 Cambridge Colorectal Unit, Addenbrooke's Hospital, Cambridge University Hospitals NHS Foundation Trust, Cambridge CB20QQ, UK

**Keywords:** quiescence, stem cell, cancer stem cell, intestine, colorectal cancer

## Abstract

Stem cells are responsible for maintaining differentiated cell numbers during normal physiology and at times of tissue stress. They have the unique capabilities of proliferation, self-renewal, clonogenicity and multi-potentiality. It is a widely held belief that stem-like cells, known as cancer stem cells (CSCs), maintain tumours. The majority of currently identified intestinal stem cell populations appear to be rapidly cycling. However, quiescent stem cell populations have been suggested to exist in both normal intestinal crypts and tumours. Quiescent CSCs may have particular significance in the modern management of colorectal cancer making their identification and characterisation a priority. In this review, we discuss the current evidence surrounding the identification and microenvironmental control of stem cell populations in intestinal crypts and tumours as well as exploring the evidence supporting the existence of a quiescent stem and CSC population in the gut and other tissues.

Over the past few decades, there have been significant advances in treatment and outcome for patients with epithelial cancers as well as our understanding of the tumour-initiating populations that drive their growth. It is now widely accepted that tumour maintenance is a function of a subset of stem-like or cancer stem cells (CSCs). Cancerous cells have various strategies to evade toxicity from chemotherapy and radiotherapy, one of which is the homeostatic phenomenon of cellular quiescence. The relative contribution of quiescent and continuously dividing stem cell populations in maintaining both normal intestinal tissue and malignant colorectal tumours remains far from clear. Both populations appear to coexist in intestine. Research from other organ systems indicates that they may have separate but cooperating functions in homeostasis and at times of injury, suggesting that the dependency on quiescence *vs* rapid cycling stem populations may vary with biological and clinical contexts. In this regard, we highlight patients with rectal adenocarcinoma. Neoadjuvant chemoradiotherapy has led to apparent pathological complete response (pCR) in some cases but a proportion of these relapse. Here, we discuss the possible features that rectal CSC populations may adopt to result in this pattern of clinical outcome.

## The CSC hypothesis

All renewing tissues require stem cells to repopulate the differentiated cell pool that is lost as a result of physiological cell turnover. It has been shown that in tumours, there exist CSCs that drive tumour growth and that possess similar characteristics of proliferation, self-renewal, clonogenicity and multi-potentiality as do stem cells in normal organs. The CSC hypothesis originates from work on haematological malignancies in the first half of the 20th century that showed only a small proportion of cells from a tumour were capable of initiating further tumour growth (Furth, 1937). It was not until 1997 that Bonnet and Dick (1997) demonstrated in acute myeloid leukaemia that this phenomenon was due to CSCs rather than stochasticity in tumour cell fate. Similar observations have subsequently been shown in a variety of solid organ tumours (Al-Hajj *et al*, 2003; Hermann *et al*, 2007; O’Brien *et al*, 2007), demonstrating that only a discrete sub-population of cells have tumour-initiating capacity. It still remains unclear if these are transformed ‘normal’ stem cells that have undergone malignant change and yet retain their ‘stem’-like characteristics or, alternatively, if they are differentiated malignant cells that have re-acquired stem-like characteristics (Chaffer *et al*, 2011). These two possibilities are not mutually exclusive, and in which tumours or specific circumstances either occur is not certain. It is important to note that the CSC hypothesis does not necessarily suggest that the stem cell is the cell of origin of the tumour although this may be the case in the intestine (Barker *et al*, 2009).

While stem cells in normal tissue are generally regarded as being rare, significant debate surrounds the prevalence of CSCs in malignancies. Much of the uncertainty surrounding this issue is a consequence of the assays widely used to assess tumourigenicity. These involve transplanting a limited number of presumed CSCs into an immunocompromised mouse and then ascertaining whether a tumour can be recreated from this subset of cells. Such assays have been criticised as the ability of the donor cells to survive and grow may be significantly compromised. For example, it has been shown using cells from human melanomas that simply changing the type of immunocompromised mouse from a NOD/SCID (non-obese diabetic/severe combined immunodeficient) to a NSG (NOD/SCID *γ*) mouse raises the frequency of tumour-initiating cells from 1 in a million to 1 in 4 (Quintana *et al*, 2008). The varying estimates of CSC prevalence could also be explained by tumour clonality and/or differentiation status; Yeung *et al* (2010) recently showed using a colony forming assay and colorectal (CRC) cancer cell lines that colony forming efficiency and morphology was not simply related to CSC marker presence but also to the individual cell line and therefore tumour of origin. They demonstrated that well-differentiated cell lines produced more differentiated colonies than more aggressive, undifferentiated cell lines. The conclusion being that the tumours from which the cell lines were derived may have had widely differing CSC populations: making up almost the entirety of the tumour in the latter and being a relatively small population in more differentiated lines. This interpretation is consistent with a non-differentiating CSC clonal population becoming dominant in poorly differentiated tumours.

The identification of CSCs has been dominated by the use of cell surface markers to isolate tumour cell sub-populations and subsequently assessing their tumour-initiating capacity. Interestingly, many putative CSC markers not only appear to mark CSCs from disparate tissues but also appear to overlap significantly with normal stem cell markers (Table 1). CD24, for example, has been shown to not only mark stem cells in the normal intestine (Gracz *et al*, 2010; von Furstenberg *et al*, 2011) and lung (McQualter *et al*, 2010) but also marks CSC populations in the colon (Vermeulen *et al*, 2008; Choi *et al*, 2009), ovary (Gao *et al*, 2010) and pancreas (Yao *et al*, 2010). Although these findings suggest overlapping regulatory functions, the picture is likely far more complex. For example, CD24 marks normal mammary stem cells (Shackleton *et al*, 2006) but in combination with other cell surface markers, CD24-negative breast cancer cells are those with greatest tumour-initiating potential (Honeth *et al*, 2008). Further, although these cell surface markers can successfully isolate stem cell populations, the protein function may not be directly related to stem cell function.

**Figure 1:**
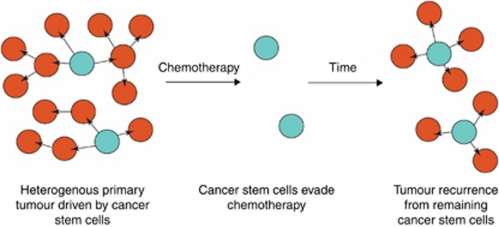
The CSC hypothesis and disease recurrence. CSCs are responsible for driving tumour growth. If CSCs rather than other malignant cells evade chemotherapy, then they can be responsible for re-establishing the tumour, clinically presenting as local recurrence or metastatic disease.

CSCs are of clinical significance as it has been shown that they are more resistant to both chemotherapy and radiotherapy than other malignant cells (Elrick *et al*, 2005; Vlashi *et al*, 2009). This may be a biological feature retained from normal tissue stem cells that natively possess various strategies to evade chemotherapy including cellular quiescence and the expression of proteins to eliminate drugs from the cytoplasm such as ABC transporters (Zhou *et al*, 2001) and MDR proteins (Terskikh *et al*, 2001). Indeed, this phenomenon of drug transport has been used by some groups to isolate stem cells based on the efflux of the Hoechst DNA binding dye. If CSCs are capable of evading adjuvant treatment then disease recurrence is likely where even a few tumour-initiating cells remain after therapy (Figure 1). Identifying, characterising and developing novel targeting strategies against CSCs should not only increase the efficacy of adjuvant therapies but also enable the identification of patients at risk of disease recurrence through poor response to treatment.

## Stem and CSC quiescence

While not being essential for stem cell function, it has been suggested that quiescence is a characteristic possessed by stem cells in many mammalian tissues (Cotsarelis *et al*, 1990; Jensen and Watt, 2006). This may be an evolutionary selected behaviour because continuous and rapid cycling is ultimately detrimental to the stem cell population as with each sequential round of cell division there increasingly exists the probability of acquiring a cumulative burden of DNA mutations. Alternatively, it has also been suggested that quiescent stem cells exist as a conditional reservoir that only become active after periods of injury where there is loss of the rapidly cycling stem cell population (Li and Clevers, 2010).

**Figure 2:**
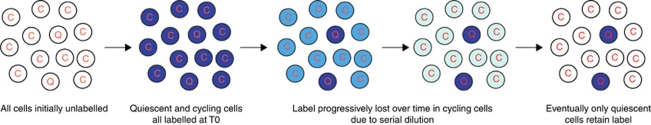
Label retaining cell studies. C=cycling cell; Q=quiescent cell. All cells are labelled with a nucleotide analogue or fluorescent reporter protein at T0. Cells that are cycling will subsequently divide thus diluting out the label. Quiescent cells retain the label enabling their isolation from the main population via FACS (fluorescence-activated cell sorting) or identification microscopically in tissue sections.

Quiescent CSCs have been isolated from melanoma (Roesch *et al*, 2010), ovarian (Gao *et al*, 2010), breast (Pece *et al*, 2010) and pancreatic tumours (Dembinski and Krauss, 2009). Many of these studies have utilised the phenomenon of ‘label retention’ in either human cell lines or mouse models to isolate putative quiescent CSCs (Figure 2). Label retaining studies involve marking all cells with a reporter protein or nucleotide analogues at a single point in time. As cells subsequently divide or die over the following days and weeks, the label is lost. Cells that are quiescent and therefore have not divided retain the label and can then be isolated for further assay. The few appropriate studies performed to date have confirmed that not only can single label retaining cells initiate tumours but they may also represent a more invasive and aggressive cell type (Mani *et al*, 2008; Pece *et al*, 2010).

Conventional chemotherapy and radiotherapy, such as is used in the treatment of rectal cancer, targets cells that are rapidly dividing. Therefore, quiescence offers CSCs a further option for evading killing. *In vitro* work in the haematopoietic system has confirmed that quiescent stem cells are less likely to be killed by cytotoxics (Cheng *et al*, 2000). There is a long standing observation of faster cell cycle times in the crypts of the distal large intestine/rectum compared with the transverse and ascending colon, presumably to some extent as a result of increased toxic and mechanical stresses (Sunter *et al*, 1979). How this impacts on the balance of quiescent and cycling stem cells is unclear but it may paradoxically generate a requirement for a higher number of quiescent stem cells. Similarly it is possible that rectal cancers may also harbour higher numbers of quiescent CSCs than other intestinal tumours.

## Colorectal stem and CSCs

**Figure 3:**
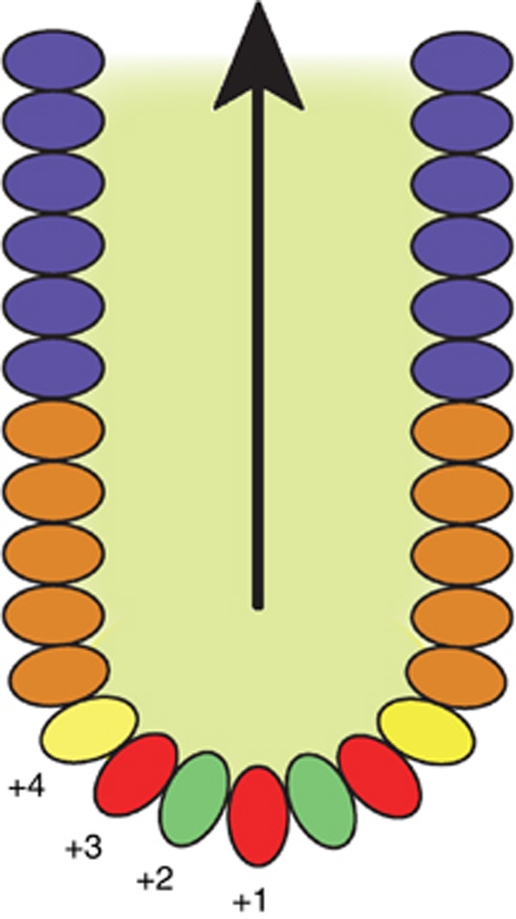
Functional arrangement of cells within small intestinal crypts. Crypts are glandular monolayer epithelial invaginations in the intestinal wall from which differentiated cells arise. In the small intestine, immunological terminally differentiated Paneth cells (red) reside in the crypt base. Interspersed between the Paneth cells are found intercalated crypt base cells (green), which are marked by Lgr5 and have been shown to be homeostatic stem cells. Above the Paneth and stem cell zone, in the +4 position, is found the location where the majority of label retaining cells (yellow) reside. Above this is known as the transit amplifying zone (brown) through which cells migrate and terminally differentiate on their way to the upper crypt (blue) and eventually the villus.

The intestine like other organs require stem cells in order to maintain adequate numbers and proportions of differentiated cells in the normal physiological state. In the small intestine, colon and rectum, these stem cells have been shown to reside in the bottom of the crypts of Lieberkûhn and are capable of driving the production of all the differentiated cell lineages of the intestine (Barker *et al*, 2007). These stem cells are not common; assuming a murine crypt population of around 250 cells, stem cells appear to comprise only 5% of this total population (Schepers *et al*, 2011). Various markers have been used to identify intestinal stem cells based in the main on the utilisation of mouse models; these include CD133 (Snippert *et al*, 2009; Zhu *et al*, 2009), CD44 (Hou *et al*, 2010), CD24 (Gracz *et al*, 2010), Bmi1 (Sangiorgi and Capecchi, 2008) and Lgr5 (Barker *et al*, 2007) (Table 2; Figure 3). Many of these identified markers have overlapping expression patterns and are often implicated in various aspects of the canonical Wnt signalling pathway, which is strongly associated with both normal intestinal stem cell function and colorectal carcinogenesis (He *et al*, 2004; Reya and Clevers, 2005; Barker *et al*, 2009; Garcia *et al*, 2009). Several of these markers, however, have problems with specificity and while overlaying stem cell populations they also mark other non-stem cells. CD24 exemplifies this issue; while having been shown to be a bona fide stem cell marker in one report, an apparently conflicting account also shows that CD24 is a marker of Paneth cells (Sato *et al*, 2011; von Furstenberg *et al*, 2011). Careful appraisal of these papers shows that CD24 has variable expression levels; while CD24^Low/Mid^ marks the intestinal stem cell compartment, CD24^High^ marks Paneth and enteroendocrine cells. Of all the markers described to date, Lgr5 has been shown to unequivocally and specifically mark the intestinal stem cell compartment as demonstrated through *in vitro* culture and *in vivo* lineage tracing studies (Barker *et al*, 2007).

Many normal intestinal stem cell markers also mark CSCs. Lgr5-positive cells have been shown to be representative of the cell of origin of intestinal tumourigenesis and have tumour-initiating potential (Barker *et al*, 2009). The degree of expression of this protein appears to relate to disease recurrence after treatment with curative intent in CRC (Merlos-Suarez *et al*, 2011). CD133 marks a group of cells that have tumour-initiating capacity at a greater level than CD133-negative cells (O’Brien *et al*, 2007). Furthermore, CD133 and CD24 expression have also been shown to relate to the degree of differentiation and invasiveness of CRC (Choi *et al*, 2009). However, the picture has become complicated by a study showing that loss rather than gain of membranous expression of the CSC markers CD44, CD166 and EPCAM is associated with CRC tumour progression (Lugli *et al*, 2010). As it has not yet been demonstrated that any one, or combination of CSC markers is capable of capturing the CSC sub-population throughout the development of a tumour, it remains possible that sub-populations may be missed.

Within the normal intestinal tract, quiescent cells have been shown to exist based on label retaining experiments. Label retention, however, only identifies quiescence *per se* and does not prove ‘stemness’. Lgr5-positive cells are rapidly cycling and have been shown using a variety of approaches to have a cell cycle time approximating to around 24 h (Escobar *et al*, 2011; Schepers *et al*, 2011). Also, by analysis of clonal population dynamics, it has been shown that in the physiological situation, there can exist only one equipotent but potentially heterogeneous stem cell population (Lopez-Garcia *et al*, 2010). These data suggest that the whole intestinal stem compartment in normal physiology is rapidly cycling but they do not address the possibility of plasticity during times of injury, that is, a cell acquiring stem-like characteristics. A quiescent ‘reserve’ cell with label retaining features may represent the quiescent stem cell. Various markers including Bmi1 (Sangiorgi and Capecchi, 2008), Wip1 (Demidov *et al*, 2007), pPTEN (He *et al*, 2004), DCAMKL-1 (May *et al*, 2009) and more recently mTert (mouse telomerase reverse transcriptase) (Montgomery *et al*, 2011) have been shown to overlay the position where label retaining cells are most commonly found known as the supra-Paneth cell position +4 (the cell position from the crypt base). Debate, however, surrounds whether indeed these are truly a separate and quiescent stem cell population or an overlapping population with rapidly cycling Lgr5 stem cells. These putative quiescent cells have in some cases been shown to be capable of clonogenic expansion *in vivo* (Sangiorgi and Capecchi, 2008). In the case of mTert cells, intriguingly this clonogenicity increases after radiation induced epithelial insult. mTert as well as being a marker, may have a significant functional role as well. Maintenance of telomeres is essential for cells to avoid senescence after repeated rounds of division and therefore increased expression of mTert would be beneficial for stem and CSCs.

The existence of quiescent colonic and rectal CSCs remains largely unexplored not in the least due to the current lack of a definitive marker. The identification of their counterparts in the normal intestine suggests an important possible role for quiescent CSCs in CRC. For example, if Tert-positive cells are present in rectal cancers and show increased stem-like behaviour after radiotherapy, then they may provide a potential explanation for both poor response to neoadjuvant chemoradiotherapy and recurrent disease.

## The role of the niche in physiology, tumourigenesis and regulation of quiescence

It is becoming increasingly evident that microenvironmental or ‘niche’ cues have an instrumental role in determining stem cell function and fate as well as CSC plasticity and tumour development. Recent developments using *in vitro* organoid culture as well as *in vivo* data have shown that both the mesenchyme and Paneth cells constitute the niche that provides intestinal stem cells with tightly co-ordinated signals to enable normal function involving Wnt, Notch and BMP pathways (He *et al*, 2004; Sato *et al*, 2009, 2011). Given the location of quiescent intestinal stem cells in the +4 position and in a different geographical location to that of Lgr5+ cells in the intercalated positions, they may be exposed to different signals. Indeed as well as providing instructions about differentiation, Wnt and BMP signalling also regulate proliferation. Modulations of these signals may have a direct effect on cell cycle times and account for the apparent quiescence seen at the +4 position. There is support for such regulation from epidermal studies, demonstrating that Wnt inhibition promotes stem cell quiescence (Nishikawa and Osawa, 2007). Given how reliant both skin and intestinal stem cells are on Wnt and BMP signalling, it is possible that markers of quiescent skin stem cell populations such as Lrig1 and the NFATs may mark quiescent counterparts in the intestine (Jensen and Watt, 2006; Horsley *et al*, 2008).

CSCs also require a niche. Modulation of Wnt signalling by myofibroblasts secreting hepatocyte growth factor has been shown to account for cancer cells’ stemness (Vermeulen *et al*, 2010). It has also been proposed that inflammation and hypoxia provide microenvironmental cues to alter tumour cell behaviour (Grivennikov *et al*, 2009; Yeung *et al*, 2011). This suggests that responses to or mediated by tumours can generate novel environments that are exploited by cancer cells. Looking at wider systems it appears that these types of cues also regulate quiescence and therefore similar mechanisms may be at play in the intestine and CRC (Hermitte *et al*, 2006). Changes in CSC microenvironment are inevitable after neoadjuvant chemoradiotherapy in rectal cancer and could have important clinical ramifications including changing the balance between quiescent and rapidly cycling CSCs.

## Clinical implications in the management of rectal cancer

Modern treatment of rectal cancer is multi-disciplinary involving several modalities. After histological confirmation of malignancy, subsequent treatment options are determined by radiological staging of both the primary tumour and the surrounding mesorectum, as well as an assessment for macroscopic metastatic spread. Small tumours that have no local or regional spread may proceed directly to surgical resection whereas more advanced tumours will receive either preoperative radiotherapy to reduce the risk of local recurrence or neoadjuvant chemoradiotherapy in order to downstage the primary tumour. This downstaging process is aimed at enabling complete surgical excision with a tumour-free circumferential resection margin. It is becoming increasingly apparent that a significant proportion of patients who receive neoadjuvant chemoradiotherapy have an excellent response and while most patients at present still proceed to surgical resection, in 15–27% there is often no residual tumour seen in the resected specimen; a phenomenon known as pCR (Maas *et al*, 2010). Whether these patients still require surgical resection of the rectum with its incumbent morbidity and mortality is currently under much debate. Several groups have looked at whether there are molecular markers or dominant signalling pathways to predict which patients will respond to neoadjuvant therapy and which will not. The Wnt and insulin signalling pathways (Spitzner *et al*, 2010), VEGF and EGFR levels (Toiyama *et al*, 2010) as well as the apoptotic index (Rodel *et al*, 2002) have all been implicated in responsiveness. It is likely however that the issue is far more complex than simply whether a tumour responds to neoadjuvant treatment or not. A recent pooled analysis of studies, comparing patients with pCR and those without, shows that while patients with pCR have a more favourable outcome there still exists a significant proportion of these patients who will succumb with either local recurrence or distant metastases (Maas *et al*, 2010). In patients with pCR, this study showed 5-year disease-free survival was 83.3% and there was a 2.8% 5-year risk for local recurrence. Even where a tumour is seen to have completely responded on pathological staging, any surviving CSC population no matter how small and present either locally or in the form of circulating tumour cells could potentially cause local recurrence or distant metastasis. Given that, by definition, all of the local tumour population is eliminated by neoadjuvant treatment *in situations* of pCR it is likely that the tumour-initiating cell population that is responsible for disease recurrence will be a very small population when compared with the total tumour population. Existing CSC markers tend to mark significant areas of a primary tumour volume that probably represent expansion of a dominant clone (Choi *et al*, 2009; Snippert *et al*, 2009; Lugli *et al*, 2010). A rare population of quiescent CSCs have the prevalence and biological behaviour to account for this pattern of disease recurrence.

Aside from isolation and characterisation of quiescent CSCs, there are several areas that could form the focus for further research. First, if one could identify rectal tumours that had significant populations of chemoradiotherapy-resistant cells present before neoadjuvant therapy was given, then it might make subsequent patient selection for surgical resection simpler. While many CSC populations are inherently resistant to chemotherapy and radiotherapy the histological or imaging directed identification of a putative quiescent CSC population within rectal tumours should form one avenue for future studies.

Obviously, the development of drugs to target quiescent CSCs will be of utmost importance should they be shown to have tumourigenic capacity. As well as cell-specific targeting, drugs that modulate niche signalling may be helpful in both altering cell kinetics and rendering quiescent cells vulnerable but also in forcing quiescent CSC differentiation. However, it is not solely the development of new drugs that is going to improve survival rates. Current thinking increasingly leans towards dynamism existing within stem cell populations where the biological properties of any one stem cell can change stochastically over time. If this is representative of the situation in CSCs within rectal cancers, then it implies the importance of targeting all the differentiated cells and CSCs either rapidly cycling or quiescent in the tumour at the time of therapeutic intervention concurrently by polypharmacy. Therefore, timing of chemotherapy as well as choice of drug will be of equal importance.

Based on work to date in the intestine and other tissues, the elusive quiescent intestinal stem cell is likely to exist and its presence will have significant implications not only for intestinal stem cell biology but also for the biology and treatment of rectal cancer. As rapidly cycling intestinal stem cells become increasingly well characterised, the issues of dynamism and plasticity in stem cell compartments suggests that time and resources should also be directed towards the understanding of this lesser known but equally important intestinal stem cell population.

## Figures and Tables

**Table 1:** Stem and cancer stem cell marker overlap in the major epithelia

**Marker**	**Stem cell marker**	**Species**	**Cancer stem cell marker**	**Species**
CD24	Intestine (von Furstenberg *et al*, 2011)	Mus	Intestine (Vermeulen *et al*, 2008)	Homo
	Breast (Shackleton *et al*, 2006)	Mus	Ovary (Gao *et al*, 2010)	Homo
	Lung (McQualter *et al*, 2010)	Mus	Pancreas (Yao *et al*, 2010)	Homo
	Neuronal (Pruszak *et al*, 2009)	Homo		
	Pancreas (Wang *et al*, 2005)	Mus		
CD44	Intestine (Hou *et al*, 2010)	Homo	Intestine (Dalerba *et al*, 2007)	Homo
	Prostate (Liu *et al*, 1997)	Homo	Breast (Al-Hajj *et al*, 2003)	Homo
			Pancreas (Yao *et al*, 2010)	Homo
			Prostate (Patrawala *et al*, 2006)	Homo
			Liver (Zhu *et al*, 2010)	Homo
CD133	Intestine (Snippert *et al*, 2009)	Mus	Intestine (O’Brien *et al*, 2007; Ricci-Vitiani *et al*, 2007)	Homo
	Prostate (Richardson *et al*, 2004)	Homo	Breast (Wright *et al*, 2008)	Mus
			Prostate (Collins *et al*, 2005)	Homo
			Ovary (Ferrandina *et al*, 2009)	Homo
			Brain (Singh *et al*, 2004)	Homo
			Liver (Zhu *et al*, 2010)	Homo
CD166	Intestine (Levin *et al*, 2010)	Homo	Intestine (Dalerba *et al*, 2007)	Homo
		Mus	Prostate (Rajasekhar *et al*, 2011)	Homo
Lgr5	Intestine (Barker *et al*, 2007)	Mus	Intestine (Barker *et al*, 2009)	Mus
	Skin (Jaks *et al*, 2008)	Mus		
Olfm4	Intestine (van der Flier *et al*, 2009)	Homo	Intestine (van der Flier *et al*, 2009)	Mus
Aldh1	Intestine (Huang *et al*, 2009)	Homo	Intestine (Huang *et al*, 2009)	Homo
	Breast (Ginestier *et al*, 2007)		Breast (Ginestier *et al*, 2007)	Homo
		Homo	Ovary (Kryczek *et al*, 2011)	Homo
			Lung (Liang and Shi, 2011)	Homo
Integrins	Intestine (Fujimoto *et al*, 2002)	Homo	Breast (Vassilopoulos *et al*, 2008)	Mus
	Breast (Shackleton *et al*, 2006)	Mus	Prostate (Collins *et al*, 2005)	Homo
	Neuronal (Pruszak *et al*, 2009)	Homo		
Bmi1	Intestine (Sangiorgi and Capecchi, 2008)	Mus	Brain (Bruggeman *et al*, 2007)	Mus
	Neuronal (Molofsky *et al*, 2003)	Mus	Liver (Chiba *et al*, 2008)	Homo
	Prostate (Lukacs *et al*, 2010)	Mus		
Musashi1	Intestine (Potten *et al*, 2003)	Mus		

**Table 2:** Intestinal stem and cancer stem cell markers

**Marker**	**Type of crypt cell marked**	**Proof of stemness^a^**	**Proof of cancer stemness**	**Number of cells required for tumour growth^b^**
CD24	All lower crypt cells	Organoid growth	Megacolonies Xenotransplant	N/A
CD44	Lower crypt cells except Paneth cells	Expression profile	Megacolonies Xenotransplant	200–500^c^
CD133	All lower crypt cells	*In vivo* lineage tracing	Xenotransplant	3000^c^, 262^d^
CD166	Intercalated crypt base cells and Paneth cells	Expression profile Immunofluorescence FACS	Xenotransplant	1000–4000^c^
Lgr5	Intercalated crypt base cells	*In vivo* lineage tracing	Targeted Apc deletion	N/A
Olfm4	Intercalated crypt base cells	*In situ* hybridisation	*In situ* hybridisation	N/A
Aldh1	Isolated crypt base cells	Immunofluorescence	Xenotransplant	25^e^
Integrins	All lower 1/3 crypt cells (*β*1 integrin)	Colony forming assay	N/A	N/A
Bmi1	+4 Single supra-Paneth cell	*In vivo* lineage tracing	N/A	N/A
Musashi1	Intercalated crypt base and supra-Paneth cells	Immunohistochemistry	N/A	N/A

Abbreviations: FACS=fluorescence-activated cell sorting; NOD/SCID=non-obese diabetic/severe combined immunodeficient.

a Strongest evidence quoted.

b In NOD/SCID mice.

c Dalerba *et al* (2007).

d O’Brien *et al* (2007).

e Huang *et al* (2009).
